# Exploring Contraindications for Thrombolysis: Risk of Hemorrhagic Transformation and Neurological Deterioration after Thrombolysis in Mice with Recent Ischemic Stroke and Hyperglycemia

**DOI:** 10.3390/jcm11123343

**Published:** 2022-06-10

**Authors:** Sarah Gelhard, Roxane-Isabelle Kestner, Moritz Armbrust, Helmuth Steinmetz, Christian Foerch, Ferdinand O. Bohmann

**Affiliations:** 1 Department of Neurology, Goethe University, Schleusenweg 2-16, 60528 Frankfurt am Main, Germany; roxane-isabelle.kestner@kgu.de (R.-I.K.); helmuth.steinmetz@kgu.de (H.S.); foerch@em.uni-frankfurt.de (C.F.); ferdinand.bohmann@kgu.de (F.O.B.); 2 Institute of Neurology (Edinger Institute), Goethe University, 60528 Frankfurt am Main, Germany; moritz.armbrust@kgu.de

**Keywords:** stroke, thrombolysis, hemorrhagic transformation, hyperglycemia

## Abstract

(1) Intravenous thrombolysis with recombinant tissue plasminogen activator (rt-PA) in patients with acute ischemic stroke is limited because of several contraindications. In routine clinical practice, patients with a recent stroke are typically not treated with rt-PA in case of a recurrent ischemic event. The same applies to its use in the context of pulmonary artery embolism and myocardial infarction with a recent stroke. In this translational study, we evaluated whether rt-PA treatment after experimental ischemic stroke with or without additional hyperglycemia increases the risk for hemorrhagic transformation (HT) and worsens functional outcome regarding the old infarct area. (2) In total, 72 male C57BL/6N mice were used. Ischemic stroke (index stroke) was induced by transient middle cerebral artery occlusion (tMCAO). Mice received either rt-PA or saline 24 h or 14 days after index stroke to determine whether a recent ischemic stroke predisposes to HT. In addition to otherwise healthy mice, hyperglycemic mice were analyzed to evaluate diabetes as a second risk factor for HT. Mice designated to develop hyperglycemia were pre-treated with streptozotocin. (3) The neurological outcome in rt-PA and saline-treated normoglycemic mice did not differ significantly, either at 24 h or at 14 days. In contrast, hyperglycemic mice treated with rt-PA had a significantly worse neurological outcome (at 24 h, *p* = 0.02; at 14 days, *p* = 0.03). At 24 h after rt-PA or saline treatment, HT scores differed significantly (*p* = 0.02) with the highest scores within hyperglycemic mice treated with rt-PA, where notably only small petechial hemorrhages could be detected. (4) Thrombolysis after recent ischemic stroke does not increase the risk for HT or worsen the functional outcome in otherwise healthy mice. However, hyperglycemia as a second risk factor leads to neurological deterioration after rt-PA treatment, which cannot be explained by an increase of HT alone. Direct neurotoxic effects of rt-PA may play a role.

## 1. Introduction

Stroke is a leading cause of death and disability worldwide [1,2]. The standard of care for acute ischemic stroke is intravenous thrombolysis with recombinant tissue plasminogen activator (rt-PA) [3]. However, several contraindications still limit the number of patients for whom treatment with rt-PA is an option.

In routine clinical practice, patients having had a stroke in recent history are typically not treated with rt-PA in case of a recurrent ischemic event. Although the FDA label does not list a stroke within the last three months as a strict contraindication, the most current American Heart Association (AHA) eligibility recommendations advise against the use of rt-PA in such patients [4]. This excludes a relevant proportion (2–10%) of acute ischemic stroke patients from this evidence-based therapy [5,6]. Scientific evidence for this approach is limited and mainly based on cardiological studies that showed an increased risk for intracerebral hemorrhage (ICH) in patients with recent stroke after using rt-PA to treat acute myocardial infarction [7,8]. Consequently, patients having had a stroke within the last three months were not included in randomized controlled trials evaluating the efficacy and safety of rt-PA treatment in patients with acute ischemic stroke [9].

The risk of thrombolysis in ischemic stroke patients with a history of a recent stroke has been evaluated in retrospect [10]. In a cohort study by Merkler et al., thrombolysis in recurrent stroke did not lead to an increased risk for ICH but was associated with a worse overall outcome [11]. In another study, recent asymptomatic ischemia identified on a pretreatment MRI did not increase the risk of hemorrhagic transformation (HT) [12]. Karlinski et al. analyzed the outcome of ischemic stroke in patients who received rt-PA despite contraindications from the Safe Implementation of Treatments in Stroke–Eastern Europe (SITS-EAST) registry. Of 13.007 patients, 2% were treated with rt-PA despite having had a stroke within the last 3 months. No significant difference in ICH occurrence compared to patients without a recent stroke was detected [13]. In a retrospective analysis from the “Get with the Guidelines-Stroke Register”, the authors compared the risk of ICH after thrombolysis in patients with and without a history of stroke and diabetes mellitus and did not find differences in rates of symptomatic ICH [14]. In general, patients with diabetes mellitus benefit from thrombolysis [15]. Nevertheless, data concerning the safety of thrombolysis in case of highly elevated blood glucose levels and recent stroke is still missing. 

In addition to its use in acute stroke therapy, thrombolytic therapy is also used in the context of acute myocardial infarction and pulmonary artery embolism. Current guidelines for the treatment of acute pulmonary artery embolism and acute myocardial infarction also list ischemic stroke within the last 6 months as a contraindication [16,17]. 

To the best of our knowledge, prospective studies evaluating the safety of rt-PA in patients with formal contraindications including a recent stroke and hyperglycemia do not exist. This, in particular, accounts for the combination of contraindications, where harmful effects on the blood–brain barrier may potentize. Here, translational studies allow a standardized approach to study the risk of ICH under such conditions in an unbiased way. The aim of this study was to evaluate whether thrombolysis 24 h and 14 days after an induced ischemic stroke with or without hyperglycemia has an influence on HT and the neurological outcome to mimic the scenario of thrombolysis after recent cerebral ischemia in the case of an acute ischemic event, such as a pulmonary artery embolism, a recurrent cerebral infarction, or a myocardial infarction.

## 2. Materials and Methods

### 2.1. Animals

In total, 72 male C57BL/6 mice were used (10–12 weeks, mean 24.3 g, Charles River laboratory, Sulzbach, Germany). Male animals were preferred in particular because of lower hormonal fluctuations and thus more uniform groups. All experiments were prospectively approved by the research ethics committee and continuously monitored (Regierungspraesidium Darmstadt, approval number FU/1040). Mice were housed under a 12 h light/dark cycle, held in groups up to 5 animals per cage and received water and food ad libitum. All surgery was performed under 2% isoflurane anesthesia and buprenorphine analgesia (10 mg/kg body weight). Mice were euthanized by transcardial perfusion under 4% isoflurane anesthesia. We conducted the study under consideration of the ARRIVE guidelines [18]. All staff was trained in animal care (experimental animal science certificate FELASA-B).

### 2.2. Experimental Design

**Figure 1:**
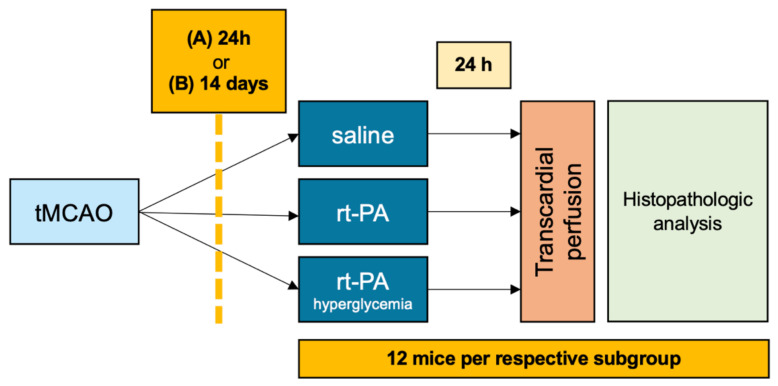
**Timeline diagram of the experimental procedures**. Hyperglycemia was induced by streptozotocin (STZ) injection 4 weeks prior to ischemic stroke. Experimental stroke was induced with transient middle cerebral artery occlusion (tMCAO) for 30 min. Saline, as control, or recombinant tissue plasminogen activator (rt-PA) was administered (A) 24 h or (B) 14 days after tMCAO.

This translational study was structured into two substudies with three arms each. Mice were allocated to the treatment groups in a randomized way by a computer-generated list, and all scientists involved in the surgical procedures and outcome evaluation were fully blinded regarding the group assignment. In the first substudy, normoglycemic mice received either rt-PA (first arm of the substudy) or saline as control (second arm of the substudy) 24 h after transient middle cerebral artery occlusion (tMCAO, index event), to simulate thrombolysis after a very recent ischemic stroke. In the third arm of the substudy, streptozotocin (STZ) pretreated hyperglycemic mice received rt-PA 24 h after tMCAO to analyze if hyperglycemia as a second risk factor increases the risk of ICH after recent stroke and thrombolysis (Figure 1). The aim of the experimental setup was to assess the extent of hemorrhagic transformation as well as neurological deterioration with respect to the index event; therefore, no new stroke was induced.

In the second substudy, we used the same experimental design as described above, but rt-PA/control was administered 14 days instead of 24 h after tMCAO (Figure 1). A second time point was chosen to determine at what point thrombolysis is safe despite having had an ischemic stroke. Animals observed for 14 days after tMCAO were supported with oral feeding as described previously [19].

If any of the following symptoms were present, the experiment was terminated, and the animals were euthanized within 5 min: intraoperative unstoppable hemorrhage, injury to vital organs, postoperative apathy, aggressiveness as a sign of severe pain, noticeable respiratory problems, strongly reduced feed or water intake or weight loss of >20% of the initial body weight or blood loss with exertion (Table 1). Twice a day animal health and behavior were monitored. Mice that died spontaneously within the observation period underwent a complete autopsy.

### 2.3. Sample Size Calculation

Based on the effect size of our previous studies (Cohens D = 1.75) at least 8 mice per group were needed to detect a significant difference in neurological deficit between the treatment regimens with a power of 80% and a type 1 error of 0.05 [20]. Anticipating equal or smaller differences, we randomized 12 mice per group.

### 2.4. Transient Middle Cerebral Artery Occlusion

Experimental ischemic stroke was induced by tMCAO as described previously [21,22]. All surgery was performed under 2% isoflurane anesthesia, buprenorphine analgesia (10 mg/kg body weight) and local analgesia with lidocaine 2% as described elsewhere [22]. Based on pre-experimental data evaluating ischemic lesion size, we chose a moderate ischemic lesion size of 68.6 ± 11.9 mm^3^ induced by 30-min occlusion time. For doing so, the right middle cerebral artery was occluded with filaments (6-0 medium MCAO suture, diameter 0.09–0.11 mm, length 20 mm: diameter with coating 0.22 ± 0.02 mm, Doccol Corporation, Redlands, CA, USA). Rt-PA (0.9 mg/kg bodyweight, Actilyse™, Boehringer Ingelheim, Ingelheim, Germany) or saline (250 µL) were administered intravenously as described previously [23].

### 2.5. Inducing Hyperglycemia

Hyperglycemia was induced by low-dose streptozotocin application (Sigma Aldrich, Schnelldorf, Germany) 4 weeks prior to tMCAO. STZ was solubilized in a citrate buffer (pH 4.5), and all animals received 50 mg/kg bodyweight per day for 5 consecutive days intraperitoneally as described previously [24]. Before STZ application, mice were without food for 6 h but with free access to water. Apart from that, mice had unlimited access to food [25]. Blood glucose was monitored regularly by puncturing the buccal vein. STZ application led to hyperglycemia in all mice with an average blood glucose level of 21.5 ± 5.72 mmol/L (*n* = 13) directly prior to rt-PA/saline treatment. Mice had an average blood glucose level of 18.5 ± 4.63 mmol/L 7 days after STZ application, 23.66 ± 5.21 mmol/L after 14 days and 24.99 ± 6.06 mmol/L after 21 days (*n* = 13). In comparison, mice without STZ treatment had an average blood glucose level of 8.44 ± 0.89 mmol/L (*n* = 13).

### 2.6. Quantifying the Neurological Deficit

To evaluate neurological deficit related to ischemia, the experimental Stroke Scale (eSS) was used as described previously [19]. The score ranges from 0 to 42 points and includes the following criteria: body symmetry, gait, climbing, circling behavior, forelimb symmetry, hindlimb symmetry, compulsory circling, whisker response, trunk flexion, forelimb motility, forelimb sensation, posture and beam balance test. The neurological deficit was measured before and 24 h after rt-PA/saline application. All scores were evaluated by a blinded rater.

### 2.7. Evaluation of Ischemic Lesion Size

To evaluate ischemic lesion size, animal brains were cut into 1 mm slices and stained in 2% 2,3,5-triphenyltetrazolium chloride (TTC, Merck KgaA, Darmstadt, Germany), which ensures differentiation between vital and non-vital brain tissue by staining vital mitochondria. Lesion size as well as ipsi- and contralateral hemispheres were measured by National Institutes of Health Image J software and corrected for edema as described previously [22].

### 2.8. Histopathological Analysis

For the histopathological analysis, brains were fixed in 4% paraformaldehyde, embedded in paraffin and cut into coronal blocks. Sections 3-µm thick were cut from each brain in the area of the ischemic lesion. For each staining, three sections representing the main stroke area were analyzed.

For primary outcome evaluation, hematoxylin and eosin (HE) staining was performed. To detect even small hemorrhagic lesions, we used semiquantitative histopathological analysis on the HE stained sections. To determine HT, a 5-point microscopic score was used (0 = no hemorrhage, 1 = single petechial hemorrhage, 2 = confluent petechial hemorrhage, 3 = single space occupying hemorrhage, 4 = various space occupying hemorrhage) [26,27]. HE stained sections were also evaluated to differentiate between earlier (acute) and late (subacute) stroke stages with proof of resorptive processes. For reconfirmation of histopathological results, additional photometric hemoglobin assay was performed as previously described [28].

To detect older hemorrhages in the 14-day substudy, Perl’s Prussian Blue staining for iron was performed. A 3-point microscopic score (Ferritin Score) was used to determine the extent of iron deposition (0 = no iron; 1 = single iron-positive cells; 2 = various iron-positive cells).

To detect blood–brain barrier (BBB) breakdown, immunoglobulin (Ig) G- and fibrinogen-staining were performed. For immunohistochemistry analyses, the Bond^TM^ III Fully Automated IHC/ISH stainer (Leica Biosystems) was used. Within the stainer, sections were dewaxed using the Bond^TM^ Dewax Solution (Catalog #AR9222) and subsequently rehydrated in a decreasing ethanol series. A heat-induced antigen retrieval was applied to the slides which were exposed to an EDTA-based epitope retrieval solution (Epitope Retrieval Solution 2, Catalog #AR9640) and heated to 100 °C for 10 or 20 min, subject to the specific primary antibody used. Applying the Bond^TM^ Polymer Refine Detection (Catalog # DS9800), sections were applied to a peroxide block using hydrogen peroxide to quench endogenous peroxidase activity, followed by the application of either Rabbit-anti-mouse-IgG post primary or Fibrinogen antibody. A semiquantitative histopathological 4-point score was used to determine BBB dysfunction (0 = negative for fibrinogen/IgG; 1 = weakly positive for fibrinogen/IgG; 2 = intermediately positive for fibrinogen/IgG; 3 = positive for fibrinogen/IgG). All analyses were performed by a blinded rater using a standard light microscope. Principles for valid histopathologic scoring in research were considered to rise the quality of our data [29].

### 2.9. Statistical Analyses

Graph Pad Prism 9 (Graph Pad Software Inc., La Jolla, CA, USA) was used for statistical analysis. Statistical significance for semiquantitative histopathological analysis and functional outcome in the three-armed substudies was assessed with the Kruskal–Wallis test. To compare subgroups, Dunn’s multiple comparison test was used. To detect scores that are deviating from the remaining data, Grubb’s outlier analysis (α = 0.05) was performed. The functional outcome and HT data were depicted in scatter plots and median and interquartile range (IQR) were given. Mortality of mice in the 14 day substudy was illustrated with a Kaplan–Meier survival curve. Blood glucose levels were given as mean with standard deviation. For photometric hemoglobin measurement, a Mann–Whitney test was assessed because of limited sample size, and means and standard deviation were given.

## 3. Results

### 3.1. Functional Outcome and HT after Thrombolysis 24 h after Ischemic Stroke

In the first substudy (i.e., treatment 24 h after tMCAO, outcome assessment 24 h after treatment), mice assigned to the saline group (control) had a median deficit of 17.5 (IQR 14.5–20.5, *n* = 10) before treatment and a median deficit of 15.5 (IQR 13.5–17.0; *n* = 8) after treatment (*p* = 0.30). Mice assigned to the rt-PA treatment group without hyperglycemia had a median functional deficit of 19 (IQR 17.5–20.5, *n* = 9) before and 17 after treatment with rt-PA (IQR 13.5–22; *n* = 9; *p* = 0.53). Hyperglycemic mice had a median functional deficit of 22 (IQR 16.0–24.0, *n* = 9) before and 26 (IQR 20–30.5; *n* = 9) after treatment with rt-PA (*p* = 0.09).

**Figure 2:**
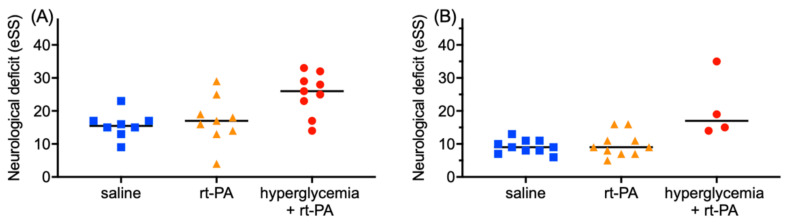
**Neurological deficit 24 h after experimental treatment**. (**A**) In the first substudy (treatment 24 h after index stroke), neurological deficit, assessed by a 42-point experimental Stroke Scale (eSS), differed significantly at the end of the observation period (*p* = 0.02). A post hoc multiple comparison test showed that hyperglycemic mice treated with rt-PA had a significantly worse neurological outcome than the saline group as control (*p* = 0.02). Comparing the neurological outcome in rt-PA and saline-treated mice did not reveal a significant difference (*p* > 0.99). (**B**) In the second substudy (14 days after index stroke), the neurological outcome differed significantly. A post hoc multiple comparison test showed that hyperglycemic mice treated with rt-PA had a significantly worse neurological outcome than the saline group as control (*p* = 0.02) and normoglycemic mice treated with rt-PA (*p* = 0.04). The values of each single mouse and the medians are depicted. Statistical significance was assessed with a Kruskal–Wallis test with Dunn’s multiple comparison test.

Comparing the three groups before treatment, functional deficit did not differ significantly (*p* = 0.19), whereas after treatment, a significant difference between the three groups could be shown (*p* = 0.02, Figure 2A). A post hoc multiple comparison test showed that hyperglycemic mice treated with rt-PA had a significantly worse neurological outcome than the control group (*p* = 0.02). Comparing the neurological outcome in rt-PA- and saline-treated mice did not reveal a significant difference (*p* > 0.99).

**Figure 3:**
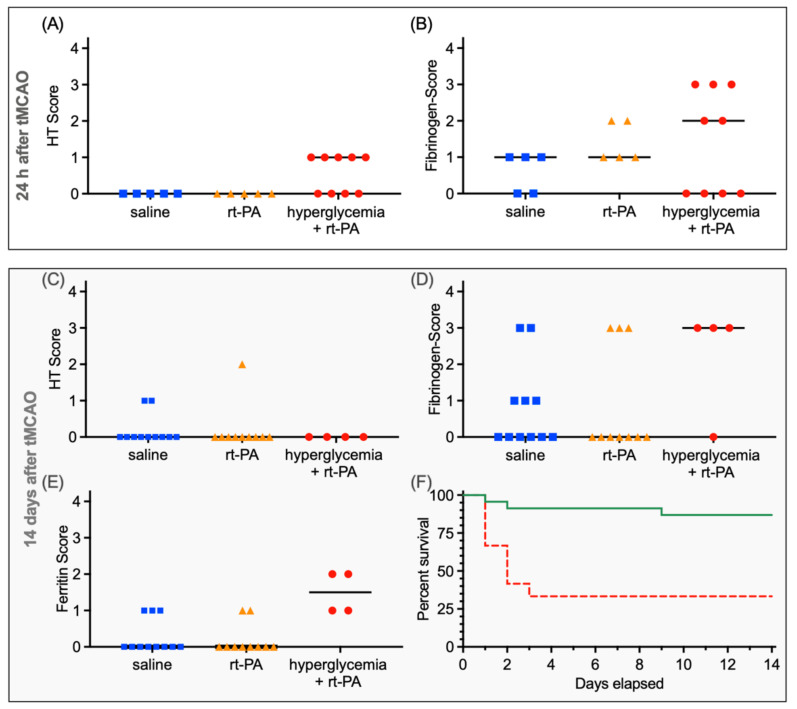
**Extent of hemorrhagic transformation (HT) and blood–brain barrier (BBB) dysfunction**. (**A**) At 24 h after rt-PA or saline treatment, the HT score differed significantly (*p* = 0.02), with the highest scores within the hyperglycemia + rt-PA group. Additionally, no significant difference in HT could be measured in photometric hemoglobin assay (saline vs. rt-PA *p* > 0.99). (**B**) Fibrinogen extravasation, reflecting the extent of BBB dysfunction, reached the highest values in the hyperglycemia + rt-PA group (*p* = 0.38). (**C**) Concerning treatment, 14 days after tMCAO the HT scores did not differ significantly (*p* = 0.66). (**D**) Fibrinogen extravasation 14 days after tMCAO did not differ significantly (*p* = 0.24). The same applies for IgG staining (data not depicted). (**E**) Nevertheless, iron staining was scored significantly higher in hyperglycemic mice (*p* < 0.01). (**F**) Mortality of mice in the 14 days substudy is illustrated in a Kaplan–Meier survival curve (red = hyperglycemic animals; green = normoglycemic animals). Importantly, due to the high mortality in the hyperglycemic group, only 4 animals could be analyzed (Hazard Ratio 11.61, CI 2.8–46.6).

At 24 h after rt-PA or saline treatment, the HT score differed significantly (*p* = 0.02, Figure 3A), with the highest scores within the hyperglycemia + rt-PA group (saline: median 0, IQR 0–0, *n* = 5 vs. rt-PA: 0, 0–0, *n* = 5 vs. hyperglycemia + rt-PA: 1, 0–1, *n* = 9). Post hoc analyses could not reveal a difference between the subgroups (saline vs. rt-PA: *p* > 0.99; rt-PA vs. hyperglycemia + rt-PA: *p* = 0.83; saline vs. hyperglycemia + rt-PA: *p* = 0.83, Figure 3). In line with the mentioned histopathological findings above, no significant difference in HT could be measured in photometric hemoglobin assay (saline vs. rt-PA *p* > 0.99, saline: 8.9 ± 4.6 µL (*n* = 5); rt-PA: 8.4 ± 5.2 µL (*n* = 5)).

### 3.2. Functional Outcome and HT after Thrombolysis 14 Days after Ischemic Stroke

In the second substudy (treatment 14 days after tMCAO, outcome assessment 24 h after treatment), the median functional deficit in the control mice was 11 (IQR 8.0–14.0, *n* = 11) before treatment and 9 (IQR 7.75–11; *n* = 10) after saline treatment (*p* = 0.25). Mice assigned to the rt-PA treatment group without hyperglycemia had a median deficit of 10 (IQR 9.0–12.5, *n* = 10) before and 9 (IQR 7–12.5; *n* = 10) after treatment with rt-PA (*p* = 0.30). Mice with hyperglycemia had a median deficit of 15 before (IQR 11.25–30.75, *n* = 4) and 17 (IQR 14.25–31, *n* = 4) after treatment with rt-PA (*p* = 0.54).

Comparing the three groups before treatment with either the rt-PA or the control functional deficit did not differ significantly (*p* = 0.13), whereas the Kruskal–Wallis test comparing neurological deficit after treatment showed a significant difference between the three groups (*p* = 0.02, Figure 2B). A post hoc multiple comparison test showed a significant difference between normoglycemic-saline-treated mice and hyperglycemic mice treated with rt-PA (*p* = 0.02) as well as between normoglycemic =-rt-PA-treated mice and hyperglycemic-rt-PA-treated mice (*p* = 0.04). Comparing the neurological outcome in rt-PA- and saline-treated normoglycemic mice did not reveal a significant difference (*p* > 0.99).

In the 14 days substudy, no significant difference between groups concerning an acute HT score were found (saline: median 0, IQR 0–1, *n* = 11, vs. rt-PA: 0, 0–0, *n* = 10 vs. hyperglycemia + rt-PA: 0, 0–0, *n* = 4, *p* = 0.66, Figure 3C).

Iron staining to detect older hemorrhages showed a significant difference (*p* < 0.01, Figure 3E) between groups with a median score of 0 in saline- (IQR 0–1, *n* = 11) and rt-PA-treated mice (IQR 0–0, *n* = 10), whereas the median in the hyperglycemia + rt-PA group was 1.5 (IQR 1–2, *n* = 4). A post hoc multiple comparison test showed a significant difference between normoglycemic-saline-treated mice and hyperglycemic mice treated with rt-PA (*p* = 0.01), as well as between rt-PA-treated normoglycemic and hyperglycemic mice (*p* = 0.01). Comparing iron deposits in rt-PA and saline treated mice did not reveal a significant difference (*p* > 0.99).

Due to the high mortality in the STZ-pretreated arm (Hazard Ratio 11.61, CI 2.8–46.6), only four animals could be analyzed here. Mortality of normoglycemic and hyperglycemic mice in the 14 days substudy was illustrated in a Kaplan–Meier survival curve (survival of hyperglycemic mice 33.33%; survival of normoglycemic mice 86.96%, Figure 3F). Due to the high mortality, the 14-day substudy was underpowered.

### 3.3. Further Histopathological Analysis

**Figure 4:**
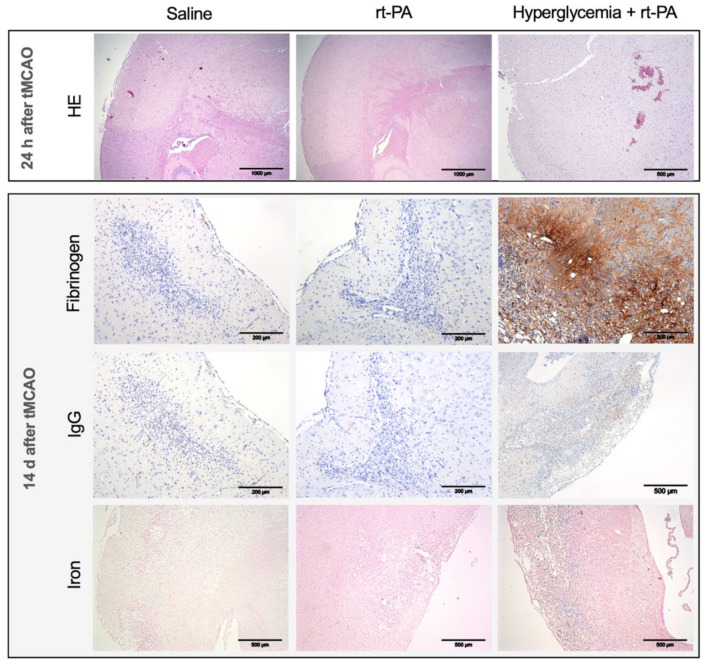
**Representative stainings for groups treated 24 h or 14 days after experimental stroke.** Depending on treatment group (rows), stainings for HE, fibrinogen, IgG and iron are presented. Sections of the right hemisphere are depicted at magnification 4× (bars 1000 µm) up to 20× (bars 200 µm). Hyperglycemic mice showed an increased extent of cerebral microhemorrhages after recombinant tissue plasminogen activator (rt-PA) treatment. Additionally, an increase of fibrinogen and immunoglobulin (Ig) G extravasation as a hallmark of blood–brain barrier breakdown could be seen sporadically in hyperglycemic mice, but a high variation of IgG and fibrinogen extravasation was detected without any difference in lesion size or localization of stroke. Statistical significance was not reached.

Transient middle cerebral artery occlusion resulted in ischemic lesions sized 68.6 ± 11.9 mm^3^ (*n* = 5) after 30 min occlusion time. Ischemic lesion volume after 2 h tMCAO was 96.0 mm^3^ (*n* = 5), and for 3 h tMCAO, it was 121.6 mm^3^ (*n* = 5, based on pre-experimental data, data not shown). Fibrinogen staining did not reveal a significant difference in BBB disruption between the three groups, neither at 24 h (saline: median 1, IQR 0–1, *n* = 5, vs. rt-PA: 1, 1–2, *n* = 5, vs. hyperglycemia + rt-PA 2: 0–3, *n* = 9; *p* = 0.38, Figure 3B) nor at 14 days after stroke (saline: 0, 0–1, *n* = 11, vs. rt-PA: 0, 0–3, *n* = 10, vs. hyperglycemia + rt-PA: 3, 0–3, *n* = 4; *p* = 0.24, Figure 3D). The same applies for IgG-staining (24 h: saline median 0, IQR 0–0, *n* = 5, vs. rt-PA: 0,0–1 *n* = 5, vs. hyperglycemia + rt-PA: 0,0–0, *n* = 9; *p* > 0.99; 14 days after stroke: saline 1, 0–1, *n* = 11, vs. rt-PA: 1, 0–1, *n* = 10, vs. hyperglycemia + rt-PA: 0.5, 0–1, *n* = 4; *p* = 0.0.87). Stainings are depicted in Figure 4. A high variation of fibrinogen detection in hyperglycemic animals was detected without any difference in lesion size or localization of stroke.

## 4. Discussion

The aim of this study was to evaluate the neurological outcome and risk of HT when rt-PA is used despite recent stroke with and without hyperglycemia. In our experimental setup, the risk with respect to the old infarct area was assessed, e.g., to evaluate the use in case of a recurrent stroke. However, these results are also of interest for thrombolysis based on pulmonary artery embolism or myocardial infarction after recent ischemic stroke.

We performed a standardized translational study based on the well-established tMCAO model to allow an unbiased assessment of the outcome parameters [30]. Occurrence of HT after rt-PA application is regularly described in this model [31]. We used an average infarct size of 68.6 ± 11.9 mm^3^ to generate moderate ischemic lesions leading to damage of the BBB and endothelial dysfunction without malignant brain swelling. We did not induce total territorial infarcts because in routine clinical practice thrombolysis would not be performed in patients with preexisting malignant media infarct.

We chose two different time points to mimic different clinical scenarios: a short-term model for acute stages of stroke (i.e., mimicking recurrent stroke within a few days after the index event) and a long-term model for resorptive stages of stroke (i.e., mimicking recurrent stroke within weeks after the index event) [32]. Thus, our model allowed assessing whether the use of rt-PA despite a recent stroke eventually becomes safer during the course of time, e.g., due to stabilization of the BBB. Since several comorbidities are usually present simultaneously in stroke patients, we added hyperglycemia as a second risk factor. Streptozotocin-induced hyperglycemia is a well-established model to depict diabetes mellitus as a cardiovascular risk factor in translational studies [33]. Our experimental study was designed to evaluate HT and the neurological outcome after thrombolysis with regard to the index stroke that has already occurred. We therefore refrained from inducing a second stroke (by a second tMCAO procedure) at the time point of rt-PA application.

In the short-term model, rt-PA did not worsen the neurological outcome in mice treated with rt-PA compared to a placebo, and there was no evidence of increased HT in rt-PA-treated mice. Those findings are in line with the existing retrospective clinical data [11]. Cell culture-based studies demonstrated that rt-PA impairs BBB predominantly via matrix metallopeptidase 9 (MMP-9) and matrix metallopeptidase 2 (MMP-2) activation [34,35]. According to our data, the threshold of BBB disturbance resulting in significant HT or a worsening of the neurological outcome is not reached in otherwise healthy mice despite a recent stroke.

It is known that hyperglycemia induces additional damage to the BBB, among others, by decreasing levels of tight junction proteins as well as by increasing MMP activity and oxidative stress [36]. In this context, hyperglycemia after ischemic stroke is well known to lead to a worse neurological outcome in animals as well as in humans. In our experimental setup, a tendency to a worse neurological outcome in the hyperglycemic subgroup was already evident before treatment with rt-PA/control, but statistical significance comparing the three groups was not reached. In addition, hyperglycemic mice showed a tendency to a worsening neurological outcome after treatment with rt-PA, even though statistical significance could not be detected in the before and after comparison. In contrast, control and normoglycemic rt-PA mice continue to recover from stroke despite the application of either rt-PA or saline. As a consequence of these opposing developments, a significant difference in direct comparison of all three groups after treatment with either rt-PA or saline could be observed.

The secondary worsening in the neurological outcome indicates that BBB damage, which is already present in hyperglycemic mice, aggravates after thrombolysis. Of note, HT differed significantly between groups, with the highest scores in hyperglycemic mice treated with rt-PA. Nevertheless, only small petechial hemorrhages were observed in hyperglycemic mice. Thus, the worsening of the neurological outcome in hyperglycemic mice after rt-PA treatment cannot be attributed to HT alone. Instead, direct neurotoxic effects of rt-PA on brain tissue may play a role. Cellular toxicity and reduced survival of neuronal cells due to rt-PA have already been described in the literature [37]. We hypothesize that rt-PA, through the disrupted BBB in the case of hyperglycemia, acts directly on cortical neurons in higher concentrations.

The results of our long-term substudy are mostly congruent with the results of our short-term substudy. In our long-term model, no worsening of neurological outcome was detected when normoglycemic animals were treated with rt-PA. HT could not be detected either.

In contrast, the neurological outcome was worse in hyperglycemic mice after rt-PA treatment. Again, saline-treated as well as rt-PA-treated mice showed further recovery despite the application of rt-PA/saline, respectively, whereas hyperglycemic mice again showed deterioration. In the before and after comparison of the respective individual groups, there is no significant difference. The improvement of normoglycemic rt-PA and saline-treated mice and the deterioration of hyperglycemic rt-PA-treated mice again resulted in a statistically significant difference when comparing all three groups post-interventionally.

Again, neurological worsening was not due to significant acute HT. Instead, subtle hemorrhages (depicted by Perls Prussian Blue staining) occurred over time after the index stroke in the hyperglycemic group. This implies a persistent BBB disorder between day 1 and day 14, again allowing rt-PA to cause additional neurotoxic effects. As a limitation of our long-term model, a mortality bias in the hyperglycemic group of the 14-day substudy must be addressed. Three animals were excluded due to weight loss without evidence of neurological deterioration, and five animals died spontaneously. In those five animals, cerebral edema due to BBB dysfunction caused by hyperglycemia followed by herniation could not be ruled out as the cause of death. Due to the high mortality. the 14-day substudy was underpowered.

Taken together, we postulate the presence of a certain threshold of BBB damage above which the risk for a worsening of the neurological outcome increases significantly after subsequent rt-PA application. The level of this threshold depends on BBB-damaging comorbidities and should be evaluated in future studies. In healthy animals, rt-PA despite recent stroke did not lead to HT or neurological deterioration.

One limitation of this study is—as discussed above—that no recurrent stroke was induced by tMCAO in the context of rt-PA or saline treatment. However, the study was designed to evaluate the risk of HT and neurological deterioration of the index stroke. This illustrates the use of thrombolysis not only in the field of stroke treatment but also in the context of pulmonary artery embolism or myocardial infarction. A limiting factor regarding the use of alteplase in the setting of pulmonary artery embolism or myocardial infarction is the deviating dosage in these indications. The effect of rt-PA on the endothelium even in the absence of an existing clot has already been demonstrated in cell cultures, and thrombolysis was applied manifold in suture occlusion models (i.e., without an existing blood clot). Therefore, no new thromboembolic event needed to be induced [23,35].

Additionally, another limiting factor is that only male mice were used. The reason for this is to keep the data variability low due to possible hormonal fluctuations in female animals and to be able to compare groups that are as homogeneous as possible. This naturally limits the transferability somewhat. Furthermore, although our results point to BBB disruption in the hyperglycemic group following the index stroke, persistent difference in BBB disruption was no longer detected at the time of sacrifice. Additionally, high variation of fibrinogen detection in hyperglycemic animals was detected without any difference in lesion size or localization of stroke. Probably, the detection method using fibrinogen and IgG was not sensitive enough, and the number of animals was too small to show a significant difference.

Moreover, no large, space-occupying HT was demonstrated in our experimental study; yet differences in the extent of HT were revealed based on microscopic evaluation using semiquantitative histopathological scores.

## 5. Conclusions

From a translational point of view, despite all limitations of transferring results from experimental studies into humans, our results suggest that in patients without additional risk factors rt-PA treatment despite recent stroke might be safe, even in the early stage (i.e., 24 h after the index stroke). However, our results indicate that additional comorbidities affecting the integrity of the blood–brain barrier may pose the risk of neurological deterioration in case of i.v. thrombolysis after recent stroke. It becomes clear that future clinical trials assessing the risk of thrombolysis after stroke should not focus on HT alone but should take neurological deterioration independent of HT into account.

## Figures and Tables

**Table 1:** Inclusion and Exclusion.

	24 h Sub-Study			14 d Sub-Study		
Experimental Group	Control	rt-PA	rt-PA + Hyperglycemia	Control	rt-PA	rt-PA + Hyperglycemia
**In total**	**12**	**12**	**12**	**12**	**12**	**12**
**Excluded mice (total)**	**3**	**3**	**3**	**1**	**2**	**8**
Excluded—Died during operation	1	2	1	0	1	0
Excluded—weight loss > 20%	0	0	1	0	0	3
Excluded—spontanously died during observation	2 *	1 *	1 *	1 *	1 *	5 *

* Mice that died spontaneously within the observation period underwent a complete autopsy. However, the autopsies showed no relevant pathology, especially no hemorrhage, ischemia or tumor.

## Data Availability

The data presented in this study are available in the article.

## Abbreviations

BBB	blood-brain-barrier
eSS	experimental Stroke Scale
HE	hematoxylin and eosin
HT	hemorrhagic transformation
ICH	intracerebral hemorrhage
IgG	immunoglobulin G
IQR	interquartile range
rt-PA	recombinant tissue plasminogen activator
STZ	streptozotocin
tMCAO	transient middle cerebral artery occlusion
